# Physiological dynamics of body temperature and heart rate during hibernation periods in male Japanese black bears (*Ursus thibetanus japonicus*)

**DOI:** 10.7717/peerj.20798

**Published:** 2026-02-17

**Authors:** Xiaofei Luo, Alice C. C. Lau, Michito Shimozuru, Mariko Sashika, Toshio Tsubota

**Affiliations:** 1 Laboratory of Wildlife Biology and Medicine, Department of Environmental Veterinary Science, Graduate School of Veterinary Medicine, Hokkaido University, Sapporo, Hokkaido, Japan; 2 Laboratory of Epidemiology, Department of Veterinary Medicine, Yamaguchi University, Yamaguchi, Japan; 3 One Health Research Center, Hokkaido University, Sapporo, Hokkaido, Japan; 4 Faculty of Veterinary Medicine, Hokkaido University, Sapporo, Hokkaido, Japan

**Keywords:** Japanese black bear, Heart rate, Hibernation, Body temperature, *Ursus thibetanus* japonicus

## Abstract

**Background:**

The Japanese black bear (*Ursus thibetanus japonicus*) exhibits a profound seasonal metabolism shift, with a substantial increase in body weight in autumn and metabolic suppression during hibernation in winter. This species hibernates under the strict regulation of endogenous and environmental factors, making it a compelling subject for studies on the interrelationships between vital signs and metabolism. Studies continuously monitoring body temperature (Tb) and heart rate (HR) in Japanese black bears is limited, leaving gaps in understanding their synchronized fluctuations during hibernation.

**Methods:**

Here, we conducted long-term monitoring to investigate changes in the Tb and HR of Japanese black bears across the pre-hibernation, hibernation-induction, hibernation, and post-hibernation periods. Subcutaneous Tb and HR loggers of six captive male bears were monitored for Tb and HR from October 2017 to May 2018.

**Results:**

The Tb of male bears decreased gradually from the pre-hibernation period to the hibernation-induction period, experienced a predicted mean Tb of 35.2 °C (95% confidence interval (CI) [35.00–35.38]) during the hibernation period, and subsequently increased gradually during the post-hibernation period. The HR decreased rapidly at the end of the pre-hibernation and hibernation-induction periods, stabilized at a predicted mean of 44.76 bpm (95% CI [34.54–54.98]) bpm during the hibernation period, and subsequently increased rapidly at the beginning of the post-hibernation period. Tb and HR followed ~24-h cycles during the pre-and post-hibernation periods. Conversely, Tb and HR fluctuations exhibited synchronization with multi-day cycles during the hibernation periods. These results suggest that Japanese black bears exhibit a daily (24-h) rhythm of Tb and HR during the pre- and post-hibernation periods, while autonomic nervous system regulation predominates during hibernation period. We observed a difference between changes in Tb and HR, Tb decreased only moderately by approximately 4% from the pre-hibernation level, whereas HR dropped dramatically by approximately 38%, with rapid changes in HR and gradual changes in Tb. Our findings enhance the understanding of hibernation physiology and highlight synchronized subcutaneous Tb and HR as key biomarkers in large hibernators.

## Introduction

Torpor, a reduction in metabolic rate, represents an adaptive response of animals to adverse environmental conditions by decreasing their metabolic activity (Hut, Dardente & Riede, 2014; Markussen et al., 2021). This phenomenon is observed in various taxa, including birds (*e.g*., swifts and hummingbirds), amphibians (*e.g*., toads), reptiles (*e.g*., lizards) (Capraro et al., 2019; Geiser, 2013; Little & Seebacher, 2014; Poo, Hinkson & Stege, 2019), and mammals (Blanco et al., 2016; Chayama et al., 2018; Currie, Kortner & Geiser, 2015; Ruf et al., 2015). Hibernation in mammals, when torpor is extended over multiple days, exhibits extreme variations in normal seasonal physiological homeostasis, particularly in terms of body temperature (Tb) (Horwitz et al., 2013). Some small mammalian hibernators, such as bats (Currie, Kortner & Geiser, 2015) and ground squirrels (Williams et al., 2011), exhibit a substantial reduction in their Tb during hibernation, decreasing from as high as 40.5 °C and 37–38 °C in bats and ground squirrels, respectively, to near ambient temperature (Ta) ranging from −11 °C to 1.5 °C. Additionally, small hibernators exhibit a reduction in Tb at the beginning of hibernation (Markussen et al., 2021), while interbout arousal, a temporary return to higher metabolic rate and Tb, occurs periodically throughout winter. Some species store food in their nests for consumption during interbout arousal.

Bears exhibit unique hibernation behaviors (Hellgren, 1998; Lundberg et al., 1976; Nelson, 1980), including prolonged periods (*i.e*., several months) of hibernation without interbout arousal, cessation of excretion, and a relatively minor reduction of ~2–5 °C in Tb. American black bears (*Ursus americanus*) hibernate for 5–7 months annually without observable interbout arousal. Additionally, they do not eat, drink, urinate, or defecate during the entire hibernation period. Brown bears (*Ursus arctos*) exhibit a Tb reduction of 5–6 °C during hibernation (Evans et al., 2016; Sahdo et al., 2013). Similarly, Japanese black bears (*Ursus thibetanus japonicus*) exhibit a Tb decrease of approximately 5 °C during the hibernation period, which remains limited (Shimozuru et al., 2016), and the changes in Tb or torpor–arousal cycles typical of small hibernators (Shimozuru et al., 2013).

Free-ranging brown bears in Scandinavia demonstrate circadian rhythms during their active state and exhibit infradian rhythms, including extended Tb and heart rate (HR) cycles exceeding 24 h, during hibernation. Evans et al. (2016) and Thiel et al. (2022) reported that brown bears initiated hibernation when snow accumulated and Ta reached 0 °C, with Tb decreasing on average 13 d prior to den entry and HR declining ahead of the Tb reduction. Hibernating black bears in interior Alaska exhibited that the Tb and HR was regulated at 30–36 °C, and substantially decreased from 55–14.4 bpm (Toien et al., 2011). Therefore, measurement of Tb and HR is essential for addressing changes in the physiological status and metabolism of hibernating bears. However, limited research exists on the simultaneous and continuous monitoring of both Tb and HR throughout the entire hibernation period remains limited and the changes in Tb and HR during hibernation in Japanese black bears are not well-understood.

Here, we aimed to elucidate the detailed patterns of subcutaneous Tb and HR changes in captive male Japanese black bears through long-term monitoring using implantable loggers during the pre-hibernation, hibernation-induction, hibernation, and post-hibernation periods. Specifically, we investigated subcutaneous Tb and HR dynamics, focusing on the patterns of decline during hibernation entry, increase during post-hibernation, and the extent of these changes. Additionally, we explored rhythmicity alterations across the pre-hibernation, hibernation-induction, hibernation, and post-hibernation periods and investigated whether subcutaneous Tb and HR fluctuated independently or synchronously during the respective periods. We hypothesized that due to the decrease in Tb and HR during hibernation, the rhythmic patterns of Tb and HR may differ from those observed in other periods. Given the scarcity of detailed physiological data on hibernation in Asiatic black bears, our primary aim was to conduct a foundational descriptive analysis of the long-term patterns and circadian rhythmicity of Tb and HR across the hibernation cycle. We establish essential baseline data and identify key physiological phenomena for future hypothesis-driven research.

## Materials and Methods

### Study animals

This study was conducted in Ani Bear Park (Kuma En) located in Kita-Akita city, Akita prefecture (39°55′N, 140°32′E), in the northern part of Japanese Honshu Island. Six adult male Japanese black bears, aged between 7 and 26 years, were selected randomly for subcutaneous Tb and HR monitoring from September or November 2017 to March or May 2018 (Tables 1 and 2). Details of the bears, including body weights before (November 2017) and after hibernation (May or June 2018), and weight loss during hibernation are listed in Table 1. For park management, male and female Japanese black bears were kept separately outdoors from late April to late November and a diet comprising mixed ingredients, mainly compressed corn was provided. All the bears were relocated to indoor enclosures devoid of light on November 23 in regular years to facilitate denning and hibernation preparation and for naturally emerging from hibernation in the spring. During the first 2 weeks of denning, the food ration was reduced to one-third of the regular amount, and then stopped until next spring (Shimozuru et al., 2013). The park typically houses two to three bears in one room with straw bedding throughout the hibernation period. During these 2 weeks, the bears would gradually reduce their activity, build their own nests with straw, and then begin to hibernate individually. This period was similar to that of the den entry mentioned in other reports (Evans et al., 2016). Although no physical barrier separating the bears existed, no interactions were observed within the same room, and each individual remained separate from the others. No food was provided during this period until the bears emerged from hibernation on April 10; however, they had unrestricted access to drinking water. As bears are anorectic and do not resume normal food intake until 10–14 d after emerging from their dens (Nelson et al., 1983), the bears were fed one-third of the active period ration for 2 weeks after the onset of feeding, and then returned to the normal amount.

**Table 1:** Age, body weight, and body mass loss of the male Japanese black bears used in this study.

Bear ID	Age (years)*	BW(Kg)		Body mass loss (%)
		November	May/June	
1	26	126	107	15.1
13	7	98	86	12.2
17	7	129	90	30.2
56	24	140	114	18.6
114	18	184	180	2.2
184	12	174	153	12.1

**Notes:**

* Age, age at the time of sampling; BW, body weight in sampling day.

Body mass loss (%) = (BW_Nov_ − BW_May/June_/BW_Nov_ * 100%).

**Table 2:** Dates and duration of subcutaneous body temperature and heart rate monitoring using implanted date loggers in male Japanese black bears.

Bear ID	Rec. start date (2017)	Anaesthetization (2018)	Rec. stop date (2018)	Total observation days
1	12 Sep.	21 Jan.	27 Mar.	197
13	13 Sep.	22 Jan.	14 May	242
17	6 Nov.	22 Jan.	14 May	190
56	10 Sep.	–	12 May	246
114	12 Sep.	20 Jan.	13 May	244
184	12 Sep.	–	13 May	244

**Note:**

Rec, recording of subcutaneous Tb and HR.

### Anesthesia and data loggers

Data loggers (data storage tag (DST) centi-HRT; StarOddi, Garðabær, Iceland) were surgically implanted subcutaneously in the thoracic region near the heart of each Japanese black bear under anesthesia. The device is small and lightweight (~19 g, 46 mm × 15 mmΦ), constructed from highly biocompatible materials. It records HR by automatically calculating from a 4 s electrocardiogram strip with a 150 Hz measurement frequency, and measures temperature ranging from 5–45 °C. Additionally, the same device was also used for other species, such as cattle and moose (Graesli et al., 2022; Palacios, Plaza & Abecia, 2021). The subcutaneous fat layer in the thoracic region was ~≤1 cm, and the logger was positioned beneath this layer, as close to the heart as possible. Anesthesia was administered *via* a blow dart using an intramuscular injection of a combination of 3.0 mg/kg zolazepam HCl and tiletamine HCl cocktail (Zoletil®, Virbac, Carros, France) and 40 μg/kg medetomidine HCl (Dorbene®; Kyoritsu Seiyaku Corporation, Tokyo, Japan) based on their estimated body weight. Body weight measurement and blood sampling (not utilized in the current study) were also conducted following implantation surgery. Benzylpenicillin potassium (penicillin potassium, 200,000 units for injection; Meiji Seika, Tokyo, Japan), meloxicam injectable solution (Metacam; Nippon Zenyaku Kogyo, Koriyama City, Japan), and atipamezole HCl (Antisedan; Kyoritsu Seiyaku Corporation, Tokyo, Japan) were administered to reduce inflammation, prevent infection, alleviate pain, and reverse sedation, respectively. Implantation was performed before the bears were transferred indoors for hibernation preparation. The implanted data loggers were programmed to record Tb and HR every 30 min. All bears (Bear IDs: 1, 13, 56, 114, and 184), comprised data loggers implanted in September 2017, except for one (Bear ID: 17), which underwent surgical implantation in November 2017. Data recording for each bear continued until March or May of the following year, as shown in Table 2. Additionally, Ta was recorded using a separate temperature sensor (iButtonsTM; Maxim Integrated Products, San Jose, CA, USA) positioned in the room where the bears were housed during hibernation. Sample size was determined based on a repeated measures design, assuming a large effect size (f = 0.4), 60–70% power, and α = 0.05. Efforts were made to use the minimum number of individuals necessary to achieve statistical significance, as well as in accordance with the permissions of the bear park. No animals were harmed or euthanized in this study. All procedures involving animals were conducted in strict accordance with the Guidelines for Proper Conduct of Animal Experiments (Science Council of Japan, 2006). The study was reviewed and approved by the Animal Care and Use Committee of Hokkaido University (Approval No.: 20-0166).

### Data analysis

The recorded data were exported using a 13-mm DST logger communication box with the Mercury software (graphic support software for DST micro-T/nano-T). We calculated a 12-h moving average of hourly data to monitor the daily changes in the subcutaneous Tb and HR of Japanese black bears. Data collected at midnight (00:00 h) were used as a representative measure of the 24-h cycle, incorporating data from both the previous and following 12 h. To mitigate the influence of blood sampling procedures on the collected data, we excluded data collected 1 week after the day of blood sampling in January for four bears (Bear IDs: 1, 13, 17, and 114), as indicated in Table 2. Based on the captive management of the bears involved here and their behavior, we defined four different observation periods: (1) pre-hibernation, the period during which bears engaged in outdoor activities until November 23; (2) hibernation-induction, the first 2 weeks following their entry into indoor enclosures, commencing on November 23; (3) hibernation, the period during which bears were inactive indoors from December to March; and (4) post-hibernation, the period during which bears awakened and resumed activities in enclosures after April 10. The daily average Tb and HR were calculated over each period: pre-hibernation (November 10–23), hibernation-induction (November 24–December 7), hibernation (December 8–April 2), and post-hibernation (April 3–May 10). After consulting with a veterinarian, we considered the first week after surgical implantation of bio-loggers as the recovery period. To evaluate changes in Tb across hibernation phases, we fitted general linear mixed models (GLMM) using the mgcv package in R (Wood, 2006). Period was included as a fixed categorical predictor, while BearID was modeled as a random effect smooth (s(BearID, bs = “re”)) to account for repeated measures within individuals. The general model form was:

${Y_{ij}} = {\rm Perio}{{\rm d}_{ij}} + s\left( {{BearI}{{ D}_i}} \right),$

${Y_{ij}}$ represents either HR or Tb of bear $i$ on day $j$.

Models were fitted by restricted maximum likelihood (REML). Model summaries were used to evaluate the significance of parametric effects (periods). Predicted mean HR and Tb values for each hibernation period were obtained from model coefficients and compared visually. Scatterplots of individual observations were overlaid with predicted period means using ggplot2 (Wickham, 2016). All analyses were conducted in R version 4.5.1 (R Core Team, 2025). Statistical significance of the main effect was assessed *via* F-tests (for general linear mixed models). Where a significant main effect was found, differences between individual periods were evaluated using Tukey’s multiple comparisons test for multiple comparisons.

Subsequently, we employed hourly data to monitor the fluctuation trends and changes in Tb and HR rhythmic patterns across multiple days within each period. The data used in this part of the analysis covered a timeline including both pre- and post-period changes. The biological periodicity of Tb and HR was detected using Acotogram J (Schmid, Helfrich-Forster & Yoshii, 2011), a software tool specifically designed for analyzing biological data, particularly rhythms in physiological variables, such as Tb and HR. Subsequently, a Lomb–Scargle periodogram was conducted on 1 month of data recorded during different periods. The Lomb–Scargle periodogram is commonly employed in time series analysis to identify periodic signals within irregularly sampled data, facilitating the detection of biological rhythms (Ruf, 1999; Thiel et al., 2022). Periodogram analysis was performed using ActogramJ (version 1.0). Rhythmicity was assessed against the null hypothesis of no periodic component. The software plots significance levels (*e.g*., *p* < 0.05) on the periodogram, derived from the theoretical distribution of Lomb-Scargle periodogram power under the assumption of pseudo-random (uncorrelated Gaussian) noise. These thresholds provide a robust criterion for distinguishing rhythmic signals from stochastic fluctuations. To further assess the robustness of this finding, we conducted bootstrapping significance tests in R version 4.5.1 (R Core Team, 2025). One-way analysis of variance was used for statistical analysis, followed by multiple comparisons for *post-hoc* analysis. Significant differences among the four periods for all bears were statistically analyzed, using predicted means and 95% confidence interval (CIs), and all results were generated using GraphPad Prism Version 10 (GraphPad Software Inc., San Diego, CA, USA). Comparisons were considered significant at *p* < 0.05. All values are represented as means ± standard deviations (SDs).

## Results

**Figure 1:**
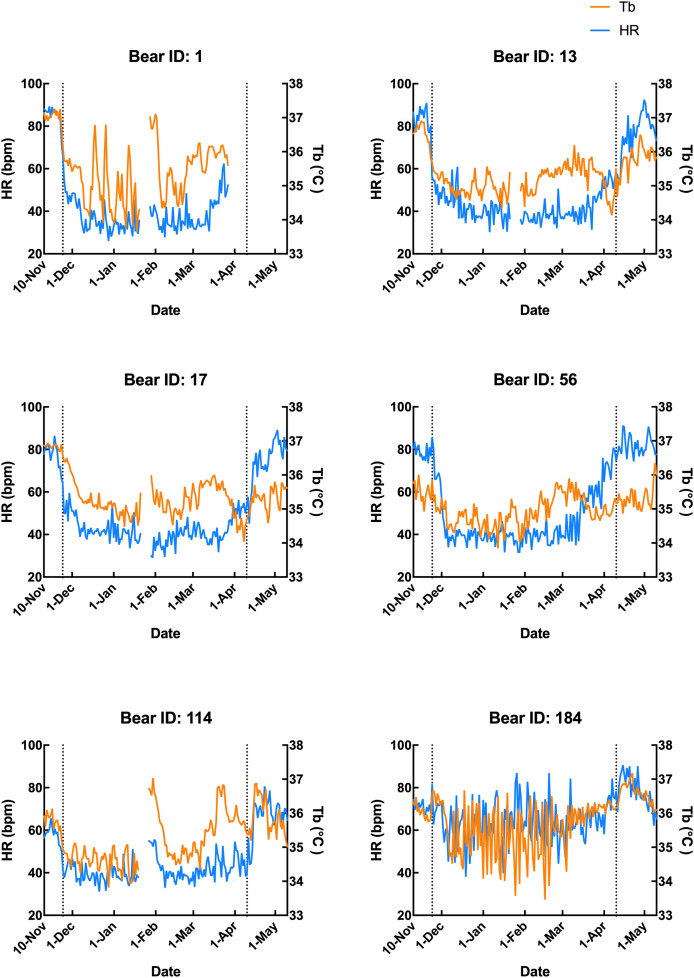
Subcutaneous body temperature (Tb; orange line) and heart rate (HR; blue line) of six Japanese black bears. Data from 1 week after the anaesthetization day was removed. The left dotted line indicates when the bears were moved indoors for denning, while the right dotted line indicates when the keeper commenced the provision of food to the bears.

**Figure 2:**
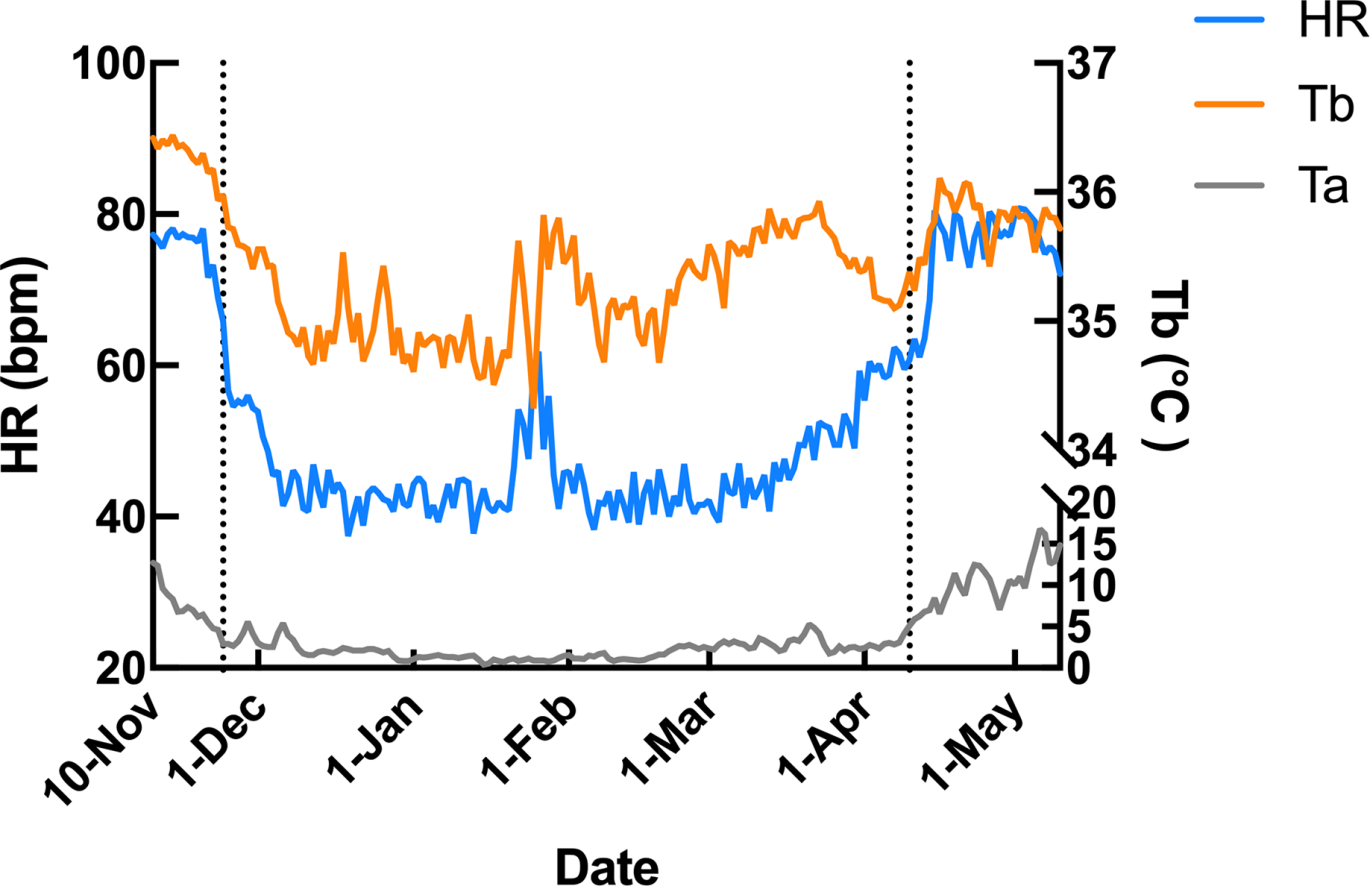
Average changes in subcutaneous body temperature (Tb; orange line) and heart rate (HR; blue line) of male Japanese black bears with ambient temperature (Ta; gray line). The left dotted line indicates when the bears were moved indoors for denning, while the right dotted line indicates when the keeper commence d the provision of food to the bears.

**Figure 3:**
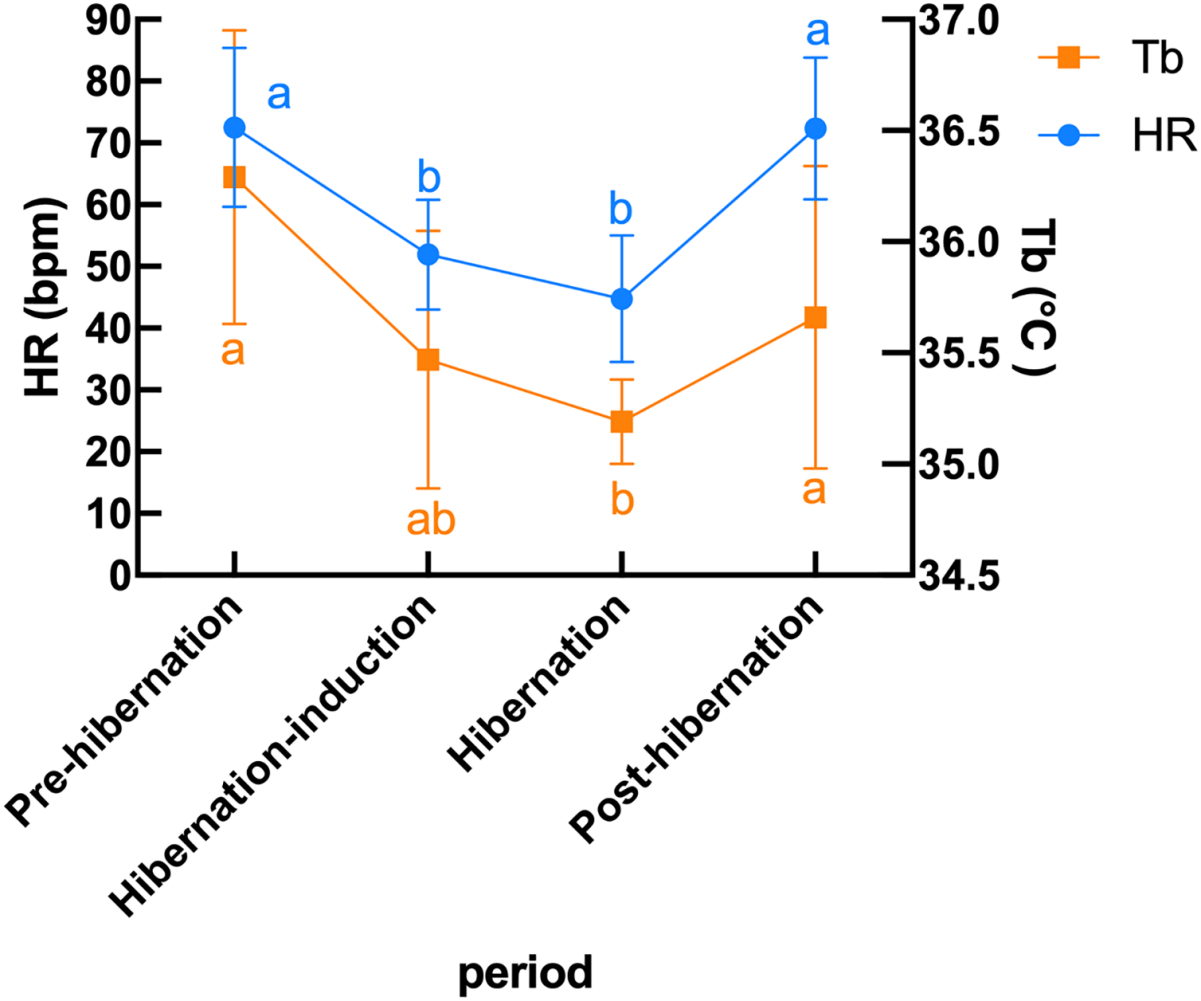
Predicted mean (± 95% CI) subcutaneous Tb and HR in six Japanese black bears during pre-hibernation, induction, hibernation, and post-hibernation periods. Data points represent predicted marginal means derived from linear mixed models, with vertical bars indicating 95% confidence intervals. Heart rate (left Y-axis, blue circles and line) showed a pronounced decline during hibernation-induction and deep hibernation periods, followed by recovery in the post-hibernation period. Body temperature (right Y-axis, orange squares and line) exhibited a smaller but significant decrease and recovery pattern. Different lowercase letters above the data points denote significant differences between periods based on Tukey’s posthoc tests (separate letters for heart rate and temperature; see Results for details).

Subcutaneous Tb and HR fluctuations varied considerably among individuals, and the variations throughout the monitoring period of six individual Japanese black bears are shown in Fig. 1. However, the average Tb and HR changes in the six bears revealed that both Tb and HR decreased significantly after den entry (hibernation-induction), remained low during hibernation, and returned to active levels in April of the following year (Fig. 2). The Ta in the den from November 10 to May 10 is depicted in Fig. 2 (gray line), with Ta falling below 5 °C from December 7 until March 21 and remaining above 0 °C throughout the entire winter period. The general linear mixed models revealed a significant primary effect of hibernation period on both body temperature (Tb) and heart rate (HR) (for both: F > 178.6, *p* < 0.001). The model-predicted marginal means and 95% confidence intervals (CIs) across periods are visualized in Fig. 3 and detailed below. For body temperature, the predicted mean during the pre-hibernation period was 36.3 °C (95% CI [35.63–36.95]), and significantly decreased to 35.5 °C (95% CI [34.89–36.05]) in the hibernation-induction period. Then further to 35.2 °C (95% CI [35.00–35.38]) during hibernation period. In the post-hibernation period, Tb significantly recovered to 35.7 °C (95% CI [34.98–36.34]). Posthoc pairwise comparisons (Tukey-adjusted) confirmed significant differences between the pre-hibernation and hibernation periods (*p* < 0.05). Contrastingly, HR underwent a dramatic reduction. The predicted mean HR dropped sharply from 72.5 bpm (95% CI [59.7–85.4]) to 51.9 bpm (95% CI [43.0–60.8]) in the pre-hibernation period and upon transfer to indoor enclosures (hibernation-induction period), respectively. It reached its nadir during deep hibernation at 44.8 bpm (95% CI [34.5–55.0]), representing a 38% decrease from pre-hibernation levels. HR subsequently recovered fully in the post-hibernation period to 72.4 bpm (95% CI [60.9–83.8]), a level statistically indistinguishable from the pre-hibernation baseline. Posthoc pairwise comparisons (Tukey-adjusted) clarified the specific differences between periods. For HR, both the hibernation-induction and deep hibernation periods were significantly lower than those of both the pre- and post-hibernation periods (all adjusted *p* < 0.05). No significant differences were detected between pre- and post-hibernation periods or between induction and deep hibernation periods (all adjusted *p* > 0.05). For Tb, the deep hibernation period was significantly lower than that of the pre-hibernation period (adjusted *p* < 0.05). However, the differences between pre-hibernation and induction periods, and between deep hibernation and post-hibernation periods, were not statistically significant (adjusted *p* = 0.05 and *p* = 0.18, respectively). These results indicate that HR declines sharply during hibernation, reaching its lowest during the hibernation period, and partially recovers during awakening. Bear ID 184 exhibited different variability in physiological parameters compared with that of other individuals.

**Figure 4:**
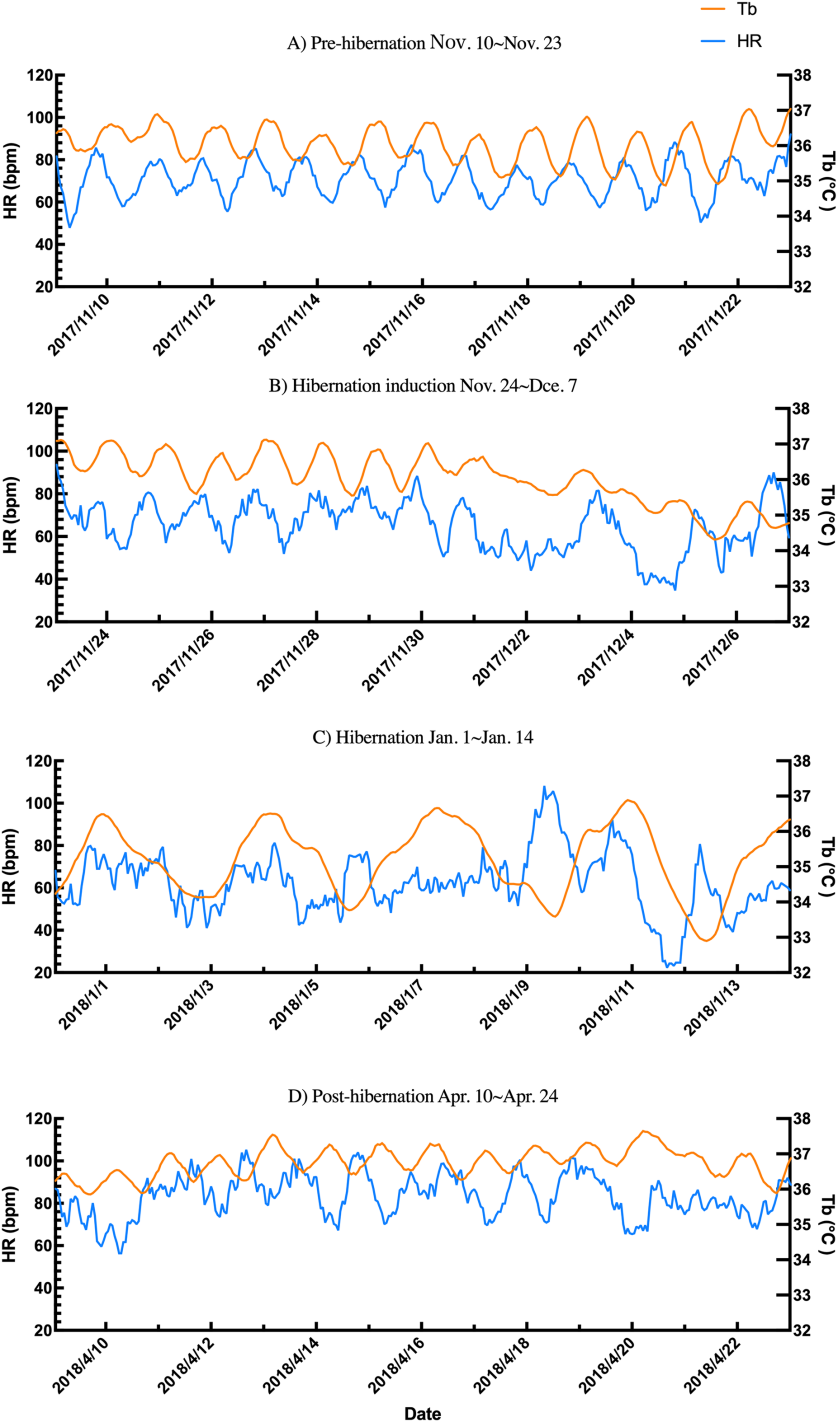
Subcutaneous body temperature (Tb; orange line) and heart rate (HR; blue line) changes of Bear ID: 56 during (A) pre-hibernation, (B) hibernation-induction, (C) hibernation, and (D) post-hibernation periods. Data for 2 weeks is shown for each period.

**Figure 5:**
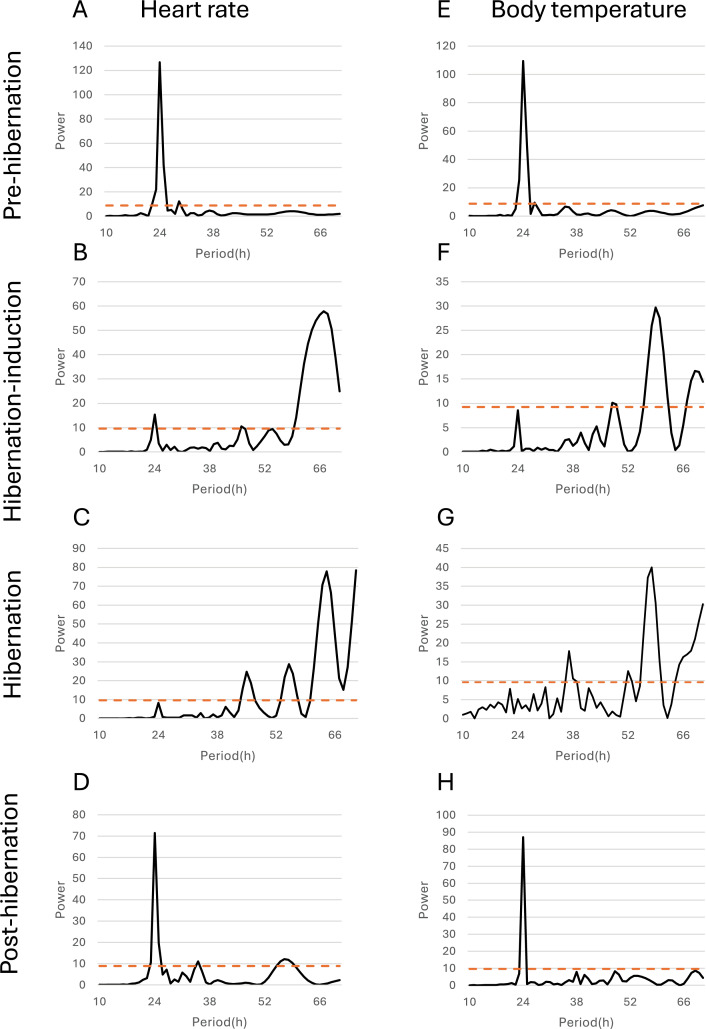
Periodicity analysis of Bear ID: 56 was conducted over 1 month, examining the 10 to 72-h periods during the pre-hibernation, hibernation, and post-hibernation periods using the same data as those in Fig. 4. Pseudo-random noise (PN) analysis included periodicity of heart rate (HR) in A, B, C, and D, and subcutaneous body temperature (Tb) in E, F, G, and H for four periods. The horizontal axis represents the period in hours (h), while the vertical axis represents the significance of the period. The dashed horizontal line indicates the *p* = 0.05 significance threshold, calculated independently for each dataset based on the false-alarm probability of the Lomb-Scargle periodogram under a pseudo-random noise null hypothesis. If the data exceeds this line, the periodicity of the observed period is considered significant.

Additionally, the relationship between changes in subcutaneous Tb and the HR for each bear was examined; an example from one bear (Bear ID 56), which did not experience any interruptions throughout the monitoring period, is presented in Fig. 4. During the pre-hibernation period, the Tb and HR of Bear ID: 56 were 34.9–36.0 °C and 73.5–83.6 bpm, respectively (Fig. 4A). Both Tb and HR displayed a 24-h cycle during the pre-hibernation period (Figs. 4A, 5A, and 5E), with Tb and HR peaking at 03:00–07:00 h and 20:00–23:00 h in Bear ID 56, respectively. The peak changes in Tb and HR exhibited a difference of several hours, a phenomenon also observed in the other five bears. Furthermore, the timing of the 24-h cycle disappearance of Tb and HR exhibited variability; specifically, the 24-h cycle in HR of Bear ID: 114 ceased on November 18, whereas the 24-h Tb cycle continued until December 3. Therefore, the Tb and HR cycles varied at different times in individual bears. During the hibernation-induction period, both Tb and HR maintained their 24-h cycles for the first few days (Fig. 4B). However, the 24-h cycles of both parameters ceased in the latter half of the hibernation-induction period (Figs. 5B and 5F). During the hibernation period, the Tb and HR varied between 32.9 °C and 36.8 °C and 34.1 to 43.4 bpm, respectively (Fig. 4C). No distinct 24-h cycle for either Tb or HR was observed, and a consistent 24-h rhythm was not evident (Figs. 5C and 5G). Instead, both Tb and HR exhibited synchronous fluctuations characterized by a multi-day cycle (Fig. 4C), and a similar pattern was observed in that of other bears, except for that in Bear ID: 17. HR increases of 1–8 h prior to Tb elevations were observed in five out of six bears during hibernation. During the post-hibernation period, Tb and HR gradually returned to normal levels observed during the pre-hibernation period (Figs. 4D, 5D, and 5H), a phenomenon also documented in other bears. The Tb and HR ranged from 34.9 °C to 35.6 °C, and 74.3 and 91.1 bpm, respectively. A 24-h cycle for Tb and HR was reestablished during this period. We detected the presence of a significant rhythm at 24 h (*p* < 0.05) using Lomb-Scargle periodograms. However, when a more conservative global maximum test was performed using bootstrapping, the rhythm failed to reach significance (*p* > 0.05), suggesting the presence of other, more strongly periodic components in the data, or that the evidence for the 24-h period is relatively weak after correction for multiple comparisons.

## Discussion

Here, we conducted long-term monitoring of subcutaneous Tb and HR in six captive male Japanese black bears across pre-hibernation, hibernation-induction, hibernation, and post-hibernation periods. We presented the Tb and HR profiles for each period, revealing a decrease in both physiological parameters during hibernation; however, the changes in Tb (a 4.1% decrease, from 36.3 °C to 34.8 °C) were relatively small compared with those in HR (a 41.3% decrease, from 72.5 to 42.5 bpm) during hibernation. Additionally, we observed that Tb and HR fluctuated synchronously with a multi-day cycle during hibernation, contrasting with those of the pre- and post-hibernation periods, which displayed a clear 24-h cycle for Tb and HR. Nevertheless, these findings contribute to our understanding of the physiological adaptations of Japanese black bears during hibernation and their temporal patterns across different periods.

The subcutaneous Tb changes in Japanese black bears during hibernation observed here were small compared with those in small hibernators. For example, the Tb of arctic ground squirrels declined from ~37.4–38.2 °C to 1.2 ± 0.3 °C upon entering hibernation (Williams et al., 2011). Similarly, the Tb of Syrian hamsters declined from 37 °C to 6 °C following the onset of hibernation (Tamura et al., 2012). However, the Tb of brown bears decreased from 37.2 ± 1.6 °C to 33.8 ± 2.1 °C (Evans et al., 2016), while the Tb of American black bears decreased from 37–38 °C to ~33 °C, indicating relatively small Tb changes, similar to those observed in Japanese black bears in this study. Additionally, we observed a considerable range of subcutaneous Tb fluctuations during the hibernation period. While some bears (Bear IDs: 13, 17, and 56) maintained a relatively stable Tb of ~34–35 °C, other bears (Bear IDs: 1, 114, and 184) displayed intermittent periods of relatively high Tb, occasionally exceeding 36.5 °C and approaching 37 °C, similar to that observed during the active period. This phenomenon was also observed in American black (Toien, Blake & Barnes, 2015) and brown bears (Evans et al., 2016), with Tb fluctuating during hibernation between 30–36 °C and 31–35 °C, respectively. Nevertheless, the daily average subcutaneous Tb of the six male Japanese black bears decreased significantly during the hibernation period, exhibiting prominent fluctuations and individual differences.

**Figure 6:**
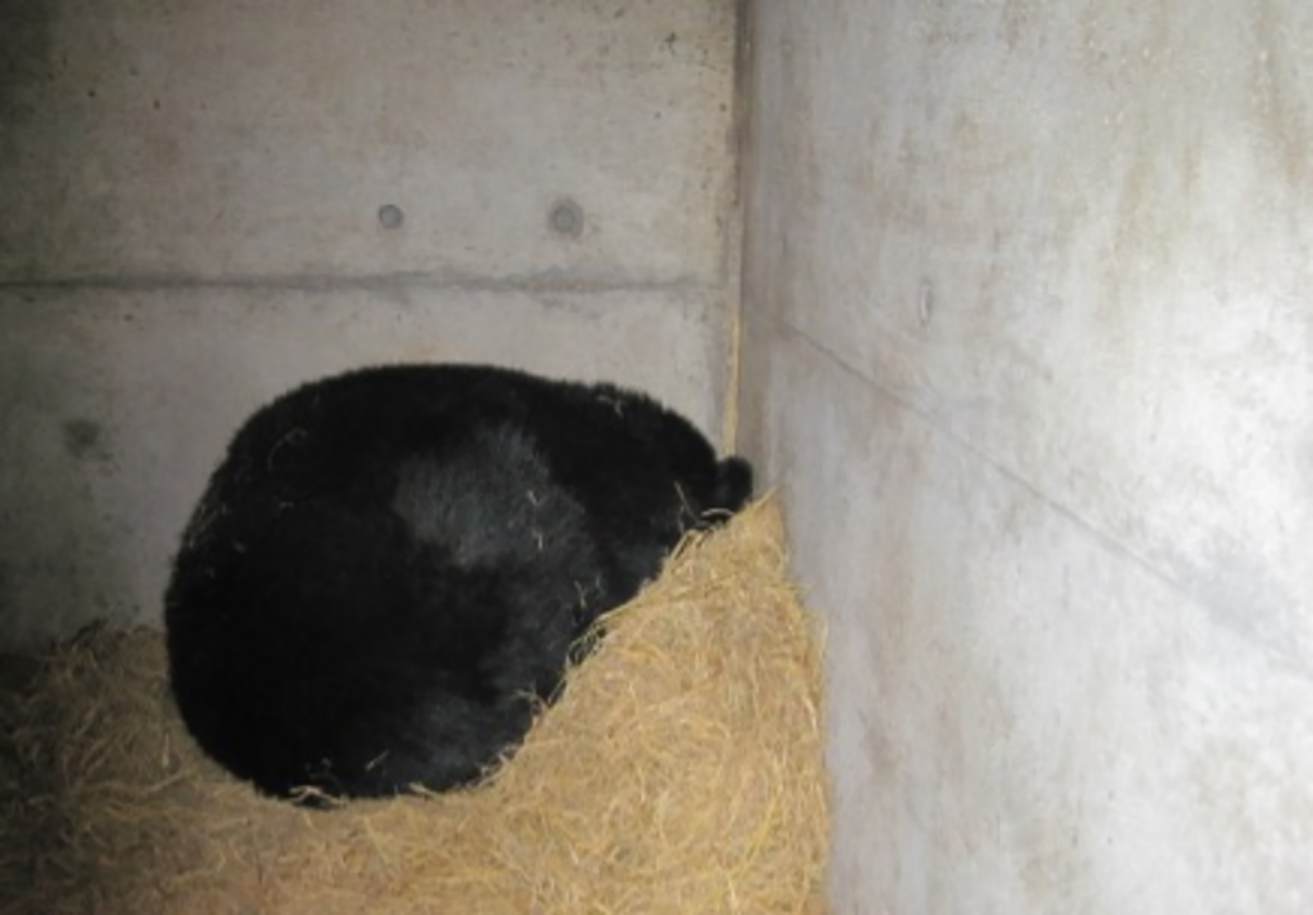
Japanese black bear during the hibernation period in Ani Bear Park.

The average Tb of bears remains relatively high (average 34.8 °C) during hibernation in our study, whereas other studies demonstrated that Japanese or American black bears exhibit Tb of ~30 °C during hibernation (Shimozuru et al., 2013; Toien, Blake & Barnes, 2015). Male Japanese black bears in the wild typically weigh ~70 kg in autumn (Oi & Furusawa, 2008); however, captive bears in this study weighed 98–184 kg, at least 30 kg more than that in an earlier study (Table 1). The well-nourished bears with their fat reserves may maintain a relatively high Tb during hibernation when adopting a curled sleeping position, along with a thick layer of straw bedding (Fig. 6). Our observation of a trend where individuals with greater body mass maintained relatively higher Tb is compatible with that of the findings from studies on free-ranging brown bears, where heavier individuals were found to maintain higher Tb throughout hibernation (Evans et al., 2023). This may indicate that the association between body condition and thermoregulatory strategy during hibernation could be a general phenomenon across bear species, potentially reflecting the greater energy reserves available to larger individuals to fuel metabolic heat production. However, this interpretation requires further validation through studies designed to directly measure the relationship between body mass and core temperature during hibernation.

In contrast to that of the changes observed in subcutaneous Tb, the HR of Japanese black bear exhibited a rapid decrease during the hibernation-induction period. The HR decreased significantly within the first 2 weeks of hibernation (hibernation-induction) and remained relatively low until the end of hibernation. This was followed by a gradual return to normal levels during the post-hibernation period (Figs. 1 and 2). The observed HR changes were consistent among all six bears from late November to mid-April. The observed consistency may indicate the synchronized indoor transfer of all captive bears, which inevitably reduces activity levels at the end of November every year. We speculate that activity reduction and a suitable sleeping environment serve as hibernation initiation cues. The decrease in HR observed in hibernating black bears correlates with metabolic suppression, as demonstrated by Toien et al. (2011), who reported the metabolic rate was reduced to 25% of the basal rate over multiple days. This reduction in metabolism decreases the demand for blood gas and nutrient transport, a phenomenon also observed in other animal species (Green, 2011). HR is closely linked to oxygen consumption rate, and hence, it directly influences the metabolic rate in most situations. Therefore, the more pronounced changes in HR observed could be attributed to metabolism suppression, although metabolic rate was not directly measured here. Similar HR patterns were observed in wild black and grizzly bears, both of which exhibit considerably slow HRs during winter dormancy (Folk & Minor, 1972). In wild American black bears, the average daytime HR ranges from 8 bpm (hibernation) to 135 bpm (summer) (Laske, Garshelis & Iaizzo, 2011). Another study on hibernating brown bears revealed a reduction in HR from 55 bpm to as low as 9 bpm, with marked variation in inter-beat intervals throughout the breathing cycle, indicating a profound sinus arrhythmia (Toien et al., 2011). Additionally, wild brown bears also showed a mean daytime HR of 16 bpm, ranging from 7–70 bpm during hibernation (Evans et al., 2023). The wild American black and brown bears in these studies showed a significantly lower HR during hibernation than that observed in our study. However, another study that monitored HR changes in wild female Japanese black bears and brown bears from summer into hibernation revealed that Japanese black bears showed similar range and temporal changes of HR as those in our study, with higher HR (110 bpm) observed during the peak hyperphagia period dropping to below 40 bpm in hibernation (Fuchs et al., 2019). Species-specific traits and nutritional condition under captivity may contribute to the physiological difference of HR. As the same model of recording device was used in both studies, the extent of HR decline during hibernation in Japanese black bears was nearly identical. The alignment of our captive results with wild data suggests that the HR reduction mechanism for hibernation is consistent across bear species.

In addition to observing overall trends, we noted periodic changes in subcutaneous Tb and HR during different periods (Fig. 5). Distinct patterns were observed in Tb and HR during the pre-hibernation, hibernation, and post-hibernation periods. During the pre-hibernation and post-hibernation periods, both Tb and HR exhibited a 24-h cycle, although their peaks did not coincide. Specifically, Tb peaked after midnight, whereas HR peaked in the evening. This is likely because periods of parasympathetic and sympathetic dominance alternate (Milsom, Zimmer & Harris, 1999). During the hibernation period, we observed a divergence in circadian patterns. Five of the six bears (IDs: 1, 13, 56, 114, 184) lost their 24-h circadian rhythm in both Tb and HR. In these individuals, the dominant periodic signal shifted to synchronized infradian (multi-day) cycles, with periods ranging from 1.6 to 7.3 days (Fig. 4B). Contrastingly, one bear (ID 17) maintained a detectable 24-h rhythm throughout hibernation, indicating notable individual variation. This phenomenon aligns with that of the observations in American black bears, where no consistent 24-h circadian rhythm in Tb was detected in January (Toien, Blake & Barnes, 2015). Similarly, another study of hibernating American black bears reported the absence of circadian rhythms in Tb (Harlow et al., 2004). Additionally, reports of brown bears exist indicating that the probability of Scandinavian brown bears showing infradian rhythms was 79% during hibernation (Thiel et al., 2022). However, these observations exhibit inconsistencies across various studies. For example, research on captive grizzly bears showed evidence of a free-running circadian rhythm during hibernation, indicating that the circadian system remains functional even when housed in constant darkness (Jansen et al., 2016). These discrepancies suggest that further research is required to determine whether circadian rhythms in Tb and HR are maintained during hibernation. While earlier studies established foundational knowledge of hibernation physiology in North American black bears, our data on Asiatic black bears provide a crucial geographical extension. Documenting conserved patterns (ranging from the persistence of a robust rhythm in Bear ID 17, to weak ~24 h rhythms in hibernation-induction period, and finally to a complete loss of rhythmicity in Bear ID: 1, 13, 56, 114 and 184) in a distinct subspecies across its range contributes to a more comprehensive understanding of bear physiology. These findings suggest that certain circadian traits may be robust across species and environments, while other physiological parameters may exhibit regional adaptations. Future studies incorporating populations from across the latitudinal and ecological gradient of the range of species will be crucial in elucidating the full spectrum of physiological adaptations in bears. An important consideration in interpreting our results is the use of subcutaneous temperature loggers. While this method is less invasive than that of abdominal implantation, it is well-established that subcutaneous temperature can be influenced by peripheral vasoconstriction/vasodilation and ambient conditions to a greater extent than that by core Tb. This may explain some of the high-frequency variability observed in our Tb traces, particularly during the active periods. During deep hibernation, when thermoregulation is minimized and the temperature gradient between the body core and shell is reduced, we expect subcutaneous temperature to be a more reliable proxy for core temperature. Our findings of attenuated 24 h rhythms are consistent with those of the studies measuring core temperature in other bear species, suggesting that the central circadian regulation of Tb is conserved across implantation sites and species (Toien, Blake & Barnes, 2015). Collectively, we believe that a strong indication exists of a 24-h rhythmic component in the data, although its intensity may not be the dominant periodic signal in the data. One reasonable explanation for the non-significance of the global test is that other strong oscillatory sources or noise in the data may exist producing peaks elsewhere in the spectrum masking the global significance of the 24-h rhythm. Therefore, although the 24-h cycle is likely to exist, caution should be exercised in interpreting it as the dominant rhythm. Future studies will need to acquire longer time series data under controlled conditions to isolate and confirm this rhythmic component.

During hibernation, an increase in HR was observed prior to elevation in subcutaneous Tb, indicating that sympathetic activation may enhance blood flow and thermogenesis to raise Tb. Conversely, HR first declined when Tb reached threshold levels, signifying decreased sympathetic activity. This cyclical process recurred throughout hibernation, establishing a multi-day cycle of Tb and HR. Similar patterns were observed in small hibernators during periodic arousal (Willis, 2007). Hibernating Arctic ground squirrels (Zanetti et al., 2023) demonstrated that HR increased 2 h prior to Tb during arousal, suggesting that sympathetic activation drives HR elevation. Studies highlighted multi-day fluctuations in Tb during hibernation without arousal, emphasizing the critical role of the sympathetic and parasympathetic nervous systems in regulating HR and Tb (Evans et al., 2016; Toien, Blake & Barnes, 2015). Heat production occurs through thermogenetic mechanisms, such as shivering (Mota & Madden, 2024) or non-shivering (Hunstiger, Johannsen & Oliver, 2023), utilizing sympathetic and parasympathetic regulation for HR and Tb changes. Black bears maintain their Tb above 30 °C primarily through shivering thermogenesis during winter (Fedorov et al., 2012; Toien, Blake & Barnes, 2015). Furthermore, although brown adipose tissue is recognized for its role in regulating Tb levels in small hibernators (Wu, Cohen & Spiegelman, 2013), no evidence supported the involvement of brown adipose tissue in bears (Gehring et al., 2016; Jones, Burnett & Zollman, 1999). In hibernating Japanese black bears, we hypothesize that parasympathetic dominance and sympathetic activation may lower Tb and HR in winter and reinstate these parameters in spring, respectively. However, the exact contribution of sympathetic and parasympathetic systems remains unclear, as the current data do not directly differentiate between these two autonomic mechanisms. During hibernation, Tb and HR may be more dependent on internal heat rather than that on Ta, suggesting that the synchronized changes in Tb and HR may reflect autonomic responses to hypothermia. Our findings are anticipated to advance our understanding of the physiological mechanisms underlying hibernation in large mammals and provide a theoretical basis for further research into autonomic and endocrine regulation during hibernation.

## Conclusions

Conclusively, we conducted a 6-month monitoring of subcutaneous Tb and HR in six captive male Japanese black bears and elucidated the dynamics of changes in both physiological parameters. Our findings revealed distinct patterns, with Tb showing gradual changes with substantial individual variation, whereas HR displayed rapid and consistent changes from the pre- to post-hibernation periods. Evident 24-h rhythms were observed in Tb and HR during the pre- and post-hibernation periods, whereas both parameters changed synchronously with time differences of the peak of HR and Tb during hibernation, suggesting their regulation by the autonomic nervous system. Future studies should explore endocrine mechanisms and physiological processes to enhance our understanding of hibernation regulation in large hibernating animals.

## Supplemental Information

10.7717/peerj.20798/supp-1 Supplemental Information 1 Average heart rate and body temperature with raw data of 6 individuals. The first and second sheets were daily average data for heart rate and body temperature of 6 Japanese black bears. The following sheets were the raw data of the heart rate and body temperature of the six bears, with an additional 24, 12, and 6 h average calculations

## Additional Information and Declarations

### Field Study Permissions

The following information was supplied relating to field study approvals (*i.e*., approving body and any reference numbers):

1. Ani bear park person in charge

Name: Sato Akihiro Tel: +810186-84-2626.

2. Akita Kitaakita city Central government office

Sightseeing promotion person in charge

Tel: +810186-62-5370.

### Data Availability

The following information was supplied regarding data availability:

The raw data is available in the Supplemental File.
